# Using Protein Painting Mass Spectrometry to Define Ligand Receptor Interaction Sites for Acetylcholine Binding Protein

**DOI:** 10.21769/BioProtoc.5163

**Published:** 2025-01-20

**Authors:** Alexandru Graur, Natalie Erickson, Nadine Kabbani

**Affiliations:** 1 School of Systems Biology, George Mason University, Fairfax, VA, USA; 2 Interdiscplinary Program in Neuroscience, George Mason University, Fairfax, VA, USA

**Keywords:** Ligand binding, Nicotinic acetylcholine receptor, Protein structure, Mass spectrometry, Drug discovery

## Abstract

Nicotinic acetylcholine receptors (nAChRs) are a family of ligand-gated ion channels expressed in nervous and non-nervous system tissue important for memory, movement, and sensory processes. The pharmacological targeting of nAChRs, using small molecules or peptides, is a promising approach for the development of compounds for the treatment of various human diseases including inflammatory and neurogenerative disorders such as Alzheimer’s disease. Using the *Aplysia californica* acetylcholine binding protein (Ac-AChBP) as an established structural surrogate for human homopentameric α7 nAChRs, we describe an innovative protein painting mass spectrometry (MS) method that can be used to identify interaction sites for various ligands at the extracellular nAChR site. We describe how the use of small molecule dyes can be optimized to uncover contact sites for ligand–protein interactions based on MS detection. Protein painting MS has been recently shown to be an effective tool for the identification of residues within Ac-AChBP involved in the binding of know ligands such as α-bungarotoxin. This strategy can be used with computational structural modeling to identify binding regions involved in drug targeting at the nAChR.

Key features

• Identify binding ligands of nicotinic receptors based on similarity with the acetylcholine binding protein.

• Can be adapted to test various ligands and binding conditions.

• Mass spectrometry identification of specific amino acid residues that contribute to protein binding.

• Can be effectively coupled to structural modeling analysis.

## Graphical overview


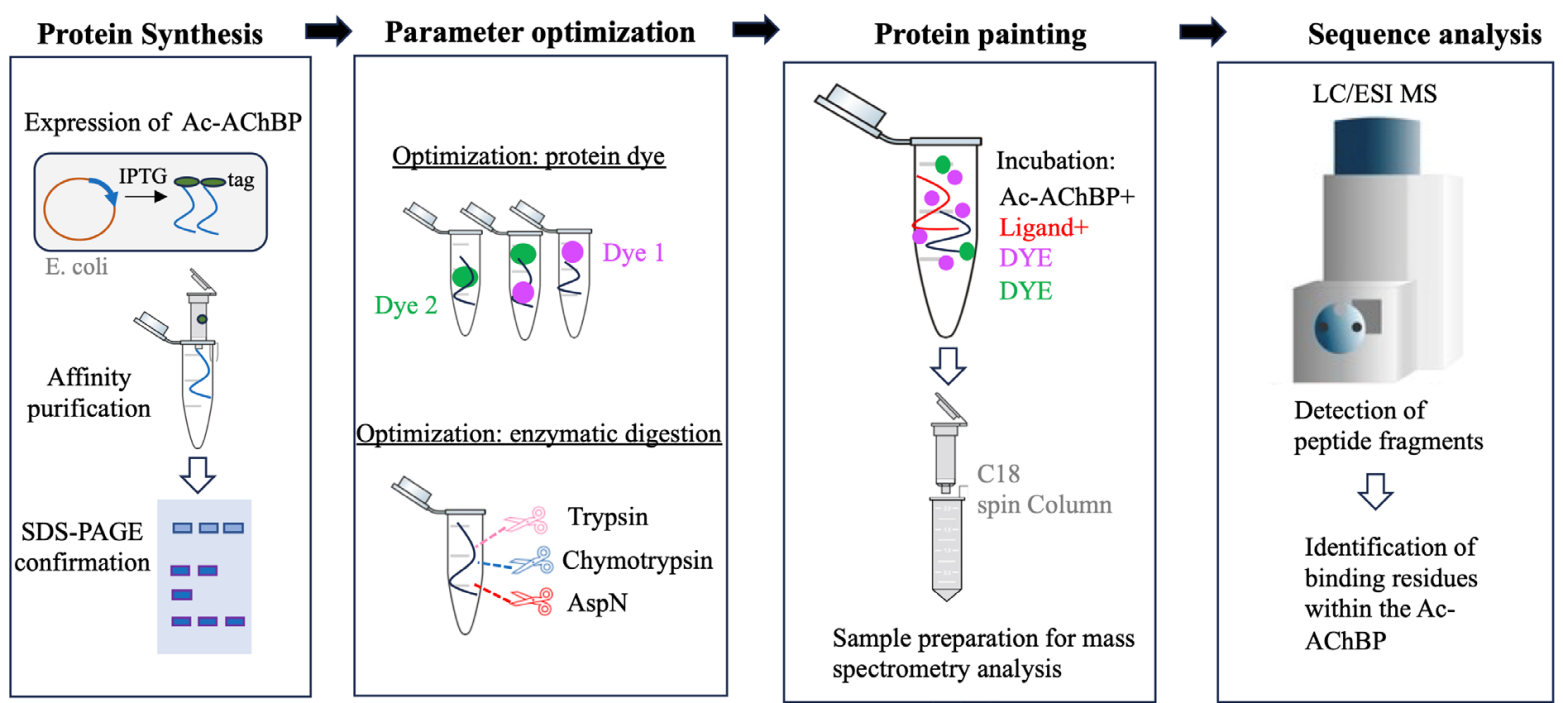


**A summary of the workflow and main steps of the protein painting experimental design.** Primary steps in the development and execution of the experiment. From left to right: The Ac-AChBP is produced in *E. coli* and then isolated using affinity chromatography; various dyes and enzymes are optimized prior to the protein paint experiment. Mass spectrometry analysis is used to define peptide fragments that are differentially produced in the painted experiment.

## Background

Mammalian nicotinic acetylcholine receptors (nAChRs) are a family of ligand ion channels, which are widely expressed in the central and peripheral nervous systems. They play key roles in modulating neurotransmitter release, synaptic plasticity, and cognitive functions; they also regulate autonomic functions by mediating fast synaptic transmission in autonomic ganglia, impacting heart rate and blood pressure [1]. The pharmacological targeting of human nAChRs is a leading strategy for therapeutic drug development in the treatment of various nervous system disorders, including Alzheimer’s and neuropathic disease [2]. Homopentameric α7 nAChRs bind important proteins including pathogenic beta amyloids and various neurotoxins [3,4]. The structural similarity, solubility, and ligand-binding properties of the homopentameric invertebrate acetylcholine binding protein (AChBP) make it a suitable model for studying ligand binding at the extracellular domain of homopentameric nAChRs [5]. In this method paper, we describe the development of a protein painting mass spectrometry (MS) approach that can serve in the detection of protein binding domains within the AChBP.

**Figure 1.**
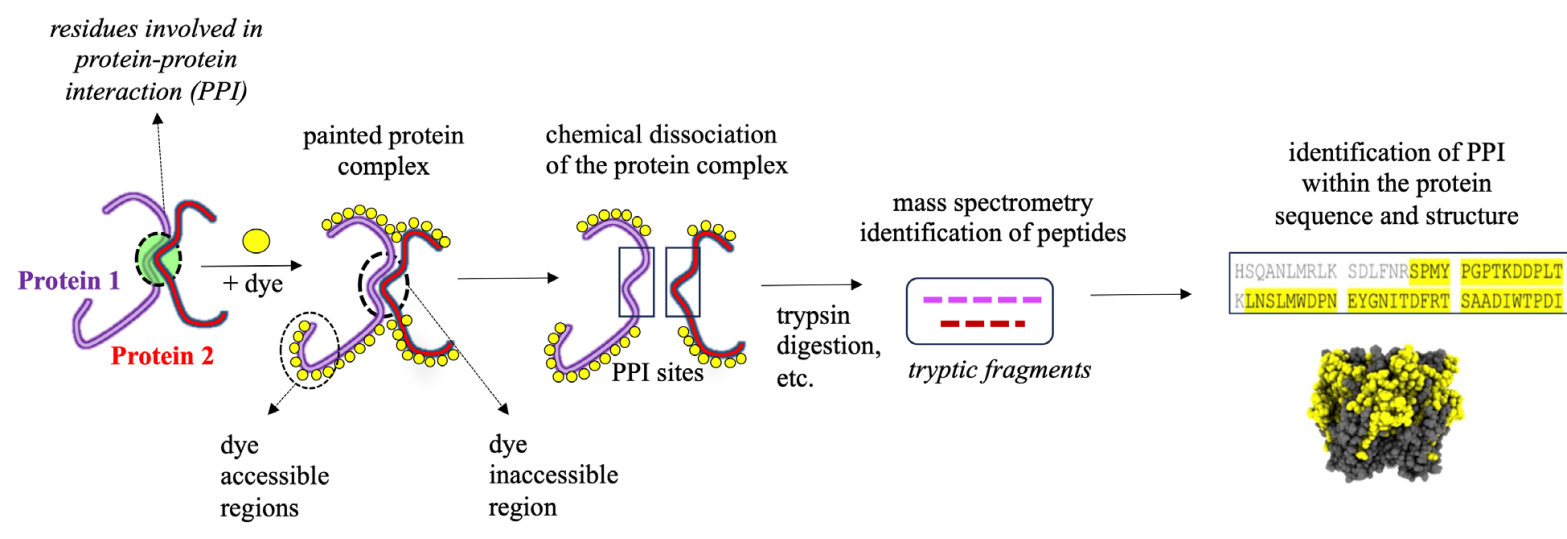
Identification of protein–protein interaction (PPI) sites through mass spectrometry (MS) protein painting. Protein painting utilizes the ability of various protein dyes to covalently bind to non-occupied (dye accessible) regions within a multi-protein complex. Dye inaccessible regions that correspond to possible PPI interaction sites are identified through MS analysis.

**Figure 2.**
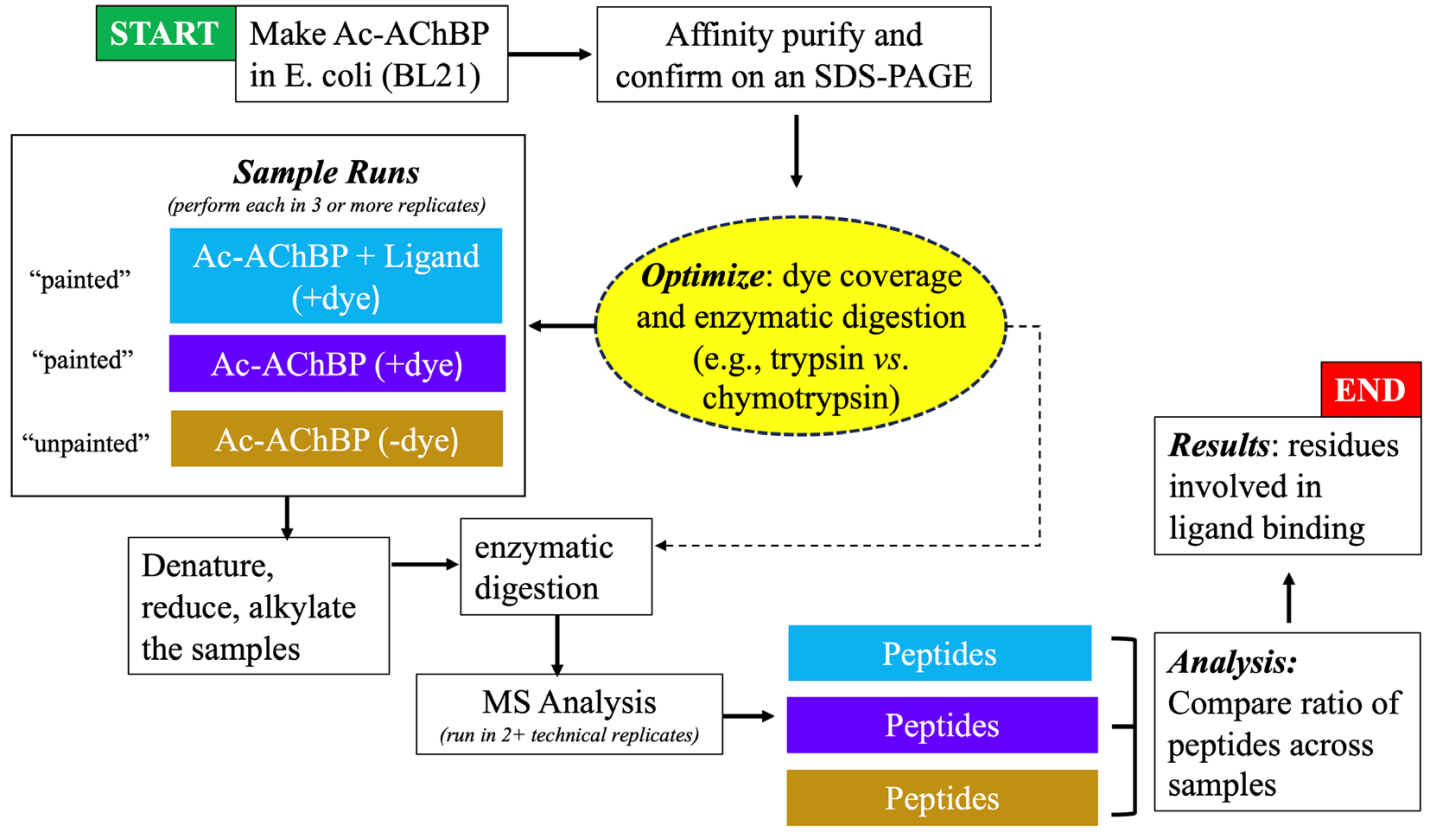
Flowchart showing the progression of experimental steps during the protein painting study

Protein painting MS technology is a new biochemical strategy in the study of ligand binding to nAChRs, recently demonstrated by our study using AChBP derived from *Aplysia californica* (Ac-AChBP) [6,7]. Protein painting employs small molecular dyes that covalently “paint” the surface of multi-protein complexes and block access to enzymatic (e.g., trypsin) digestion during MS analysis (Figure 1) [8]. Through paint coverage, this method allows for the identification of ligand-binding regions useful for drug development at the nAChR site. In addition, protein painting MS complements other experimental tools, including computational structural modeling and site-directed mutagenesis of the nAChR ligand binding pocket [9]. The protein paint technique can be easily adapted and optimized (Figure 2) on a case-by-case basis, allowing for its utility in various studies. The components of the protein paint assay are widely available and include inexpensive protein dyes, common chemical reagents, and MS instrumentation that are accessible at research institutions. Results from protein painting can guide structure-activity relationship (SAR) studies in lead compound development during drug screening and may allow for the identification of additional allosteric binding sites within the nAChR. Lastly, protein painting MS can also assess for the potential binding of new ligands to the nAChR when there is no prior knowledge of interactions.

## Materials and reagents

**Biological materials**

1. *Escherichia coli* BL21 (DE3) competent cells (Thermo Scientific, catalog number: EC0114)

**Reagents**

1. Invitrogen alpha-bungarotoxin conjugates (Thermo Fisher Scientific, catalog number: B1601)

2. Amyloid β-Protein 1-42 (Bachem, catalog number: 4014447)

3. 4-Nitrobenzenediazonium tetrafluoroborate (TCI, catalog number: N0137)

4. Disuccinimidyl suberate (DSS) (Thermo Fisher Scientific, catalog number: A39267)

5. Atto 425 NHS ester (Sigma-Aldrich, catalog number: 16805)

6. Dimethyl sulfoxide (DMSO) (Sigma-Aldrich, catalog number: D8418)

7. Anhydrous DMSO (Thermo Fisher Scientific, catalog number: D12345)

8. Choline (Acros Organics, catalog number: 110290500)

9. Nicotine (Sigma-Aldrich, catalog number: N3876)

10. Lysozyme (Thermo Fischer Scientific, catalog number: 89833)

11. Sequencing-grade trypsin (Promega, catalog number: V5111)

12. Sequencing-grade chymotrypsin (Promega, catalog number: V1061)

13. DNase (Sigma-Aldrich, catalog number: DN25-1G)

14. Complete EDTA-free mini protease inhibitor (Thermo Fisher Scientific, catalog number: A32955)

15. Protein ladder (Thermo Fischer Scientific, catalog number: 26619)

16. Phosphate-buffered saline (PBS) (VWR, catalog number: 45001-130)

17. Dithiothreitol (DTT) (Thermo Fischer Scientific, catalog number: R0861)

18. Urea (Thermo Fischer Scientific, catalog number: AC424581000)

19. Iodoacetamide (Sigma-Aldrich, catalog number: I1149)

20. Ammonium bicarbonate (Thermo Fischer Scientific, catalog number: A643)

21. Acetic acid (Thermo Fischer Scientific, catalog number: A38-500)

22. Acetonitrile (ACN) (Fischer Scientific, catalog number: A21-1)

23. Trifluoroacetic acid (TFA) (Thermo Fischer Scientific, catalog number: A116-1AMP)

24. LB broth (Thermo Fischer Scientific, catalog number: BP1426-2)

25. Ampicillin (AMP) (Thermo Fischer Scientific, catalog number: 11593027)

26. Isopropyl β-d-thiogalactopyranoside (IPTG) (Sigma-Aldrich, catalog number: I6758)

27. Tris base (Thermo Fischer Scientific, catalog number: BP152)

28. Sodium chloride (NaCl) (Thermo Fischer Scientific, catalog number: S25541)

29. Glycerol (Thermo Fischer Scientific, catalog number: J61059.AP)

30. Imidazole (Thermo Fischer Scientific, catalog number: 03196-500)

31. Triton X-100 (Sigma-Aldrich, catalog number: X100)

32. Coomassie Brilliant Blue G 250 (Sigma-Aldrich, catalog number: 1.15444)

33. Methanol (VWR, catalog number: BDH1135-4LG)

**Solutions**

1. Lysis buffer (see Recipes)

2. Resuspension buffer (Buffer A) (see Recipes)

3. Elution buffer (Buffer B) (see Recipes)

4. Final buffer (see Recipes)

5. Diazo dye (see Recipes)

6. NHS ester dye (see Recipes)

7. Sample buffer (see Recipes)

8. Coomassie solution (see Recipes)

**Recipes**

*Notes:*

*1. All buffers should be autoclaved or filtered before use and kept at 4 °C.*

*2. All dyes should be prepared fresh, immediately before use and be protected from light exposure as much as possible.*

**1. Lysis buffer**

20 mM Tris, pH 8.0

150 mM NaCl

10% glycerol

**2. Resuspension buffer (Buffer A)**

20 mM Tris, pH 8.0

150 mM NaCl

10% glycerol

10 mM imidazole

1% Triton X-100

**3. Elution buffer (Buffer B)**

20 mM Tris, pH 8.0

150 mM NaCl

10% glycerol

250 mM imidazole

1% Triton X-100

**4. Final buffer**

20 mM Tris, pH 8.0

150 mM NaCl

10% glycerol

**5. Diazo dye**

Solid 4-nitrobenzenediazonium tetrafluoroborate

1× PBS

Make 5 mg/mL stock. For each painted sample, you need 5 μL.

**6. NHS ester dye**

1 mg/mL Atto 425 NHS ester

Anhydrous DMSO

Make 5 mg/mL stock. For each painted sample, you need 5 μL.

**7. Sample buffer**

20% ACN

2% TFA

Sterile dd/diH_2_O

**8. Coomassie solution**

50% methanol

10% acetic acid

0.5% Coomassie Brilliant Blue G 250

Sterile dd/diH_2_O

**Laboratory supplies**

1. Eppendorf protein Lo-Bind 1.5 mL tubes (Eppendorf, catalog number: 022431081)

2. Sephadex G25 spin columns (Cytiva, catalog number: 27532501)

3. Pierce^TM^ C-18 spin columns (Thermo Fisher Scientific, catalog number: 89873)

4. Ni-charged IMAC column (Bio-Rad, catalog number: 12009287)

5. 30 kDa Amicon ultra centrifugal filter (Sigma-Aldrich, catalog number: UFC903008)

6. NuPAGE 4%–12% Bis-Tris gradient gel (Thermo Fisher Scientific, catalog number: NP0322BOX)

7. Graduated Erlenmeyer flasks (Sigma-Aldrich, catalog number: CLS4980250 and CLS49802L)

## Equipment

1. Centrifuge 5810R (Eppendorf, catalog number: 022625501)

2. Sorvall WX+ Ultracentrifuge (Thermo Fisher Scientific, catalog number: 75000100)

3. Water bath (Thermo Fisher Scientific, catalog number: 15-474-18)

4. Sonicator (Thermo Fisher Scientific, catalog number: 15-338-281)

5. Tube rotator (Thermo Fisher Scientific, catalog number: 13-687-12Q)

6. Spectrophotometer (Cole Parmer, catalog number: EW-83059-10)

7. Exploris Orbitrap 480 coupled with an EASY-nLC 1200 HLPC system (Thermo Fisher Scientific, catalog number: BRE725533)

8. Reverse-phase PepMap RSLC C18 LC column (Thermo Fisher Scientific, catalog number: 164534)

9. Milli-Q IQ 7003/05/10/15 water purification system (Millipore Sigma, catalog number: C205110)

10. Autoclave

11. Incubated and refrigerated console shakers (Thermo Fisher Scientific, catalog number: SHKE435HP)

## Software and datasets

1. Proteome Discoverer v2.3 (Thermo Fisher Scientific, 09/27/2019)

2. UCSF ChimeraX v1.6 (05/09/2023) (https://www.cgl.ucsf.edu/chimerax/)

3. Microsoft Excel 2016

4. RCSB Protein Data Bank (RCSB PDB) (https://www.rcsb.org/)

5. NCBI database, open access

6. Prism v10.2.0 (GraphPad, 03/26/2024)

## Procedure

**A. Make the Ac-AChBP**

**A1. Protein induction**

1. Transform *E. coli* with recombinant Ac-AChBP with the N-terminal (His)6-tag in 200 mL of LB media with AMP (100 μg/mL) selection. Grow overnight at 37 °C with shaking.

2. Inoculate the above into 1.3 L of LB media at 37 °C. Save 2 mL (out of 200 mL volume) to use as a blank for the spectrophotometer reading.

3. Monitor cell growth by measuring the OD_600_ approximately 2 h after inoculation.

4. When the absorbance reaches 0.2, add IPTG to a final concentration of 1mM to induce protein expression. Incubate at 16 °C overnight.

**A2. Cell harvest**

1. Centrifuge at 6,000× *g* for 15 min at 4 °C.

2. Discard the supernatant, resuspend the pellet in fresh LB media, and repeat the centrifugation process.

3. Discard the supernatant and keep the cell pellet.

Optional: This pellet can be frozen at -80 °C for up to four months.

**A3. Lysis and protein purification**

1. Freshly prepare the lysis, resuspension, elution, and final buffers (see Recipes).

2. Resuspend the pellet in 50 mL of lysis buffer with 500 μL of 10 mg/mL DNase, 50 mg of lysozyme, and one Complete EDTA-free mini protease inhibitor tablet.

3. Sonicate the cells and then centrifuge at 39,000× *g* for 45 min. Collect the supernatant (lysis fraction).

4. Resuspend the pellet in 50 mL of resuspension buffer (Buffer A) with one Complete EDTA-free mini protease inhibitor tablet.

5. Sonicate the cells and then centrifuge at 39,000× *g* for 30 min. Collect the supernatant (load fraction) and the pellet (pellet fraction).

6. Equilibrate the Ni-NTA column with Buffer A.

7. Wash the column with 30–50 mL of Buffer A (supernatant from step A3.5) to remove nonspecific binding proteins. Save the flowthrough (wash fraction).

8. Elute the protein with 15–20 mL of Buffer B (elution fraction).

**A4. Confirmation of protein expression**

1. Perform a Bradford assay to determine protein concentration for each fraction (pellet, lysis, load, wash, and elution).

2. Load equal amounts of protein from each fraction onto a single SDS-PAGE gel and run the gel, ensuring complete separation of the molecular weight marker bands between 60 and 10 kDa.

3. Stain the gel using a Coomassie solution stain (see manufacturer’s instructions).

4. Confirm the expression of the Ac-AChBP subunit as the major protein product on the gel. The Ac-AChBP subunit is expected to run at ~28 kDa [6].

**A5. Protein concentration and buffer exchange**

1. Concentrate the eluted protein using a 30 kDa Amicon Ultra Centrifugal filter for 15 min at 4 °C.

2. Discard the flowthrough and refill the filter with the final buffer to remove imidazole.

3. Repeat the concentration process two times to complete the buffer exchange.

**B. Protein paint**

**B1. Preparing the Ac-AChBP and ligand**

1. Determine the appropriate number of samples and label a sterile Eppendorf protein Lo-Bind 1.5 mL tube for each. For example, use one tube for every individual Ac-AChBP unpainted condition, one tube for every Ac-AChBP painted condition, and one tube for every Ac-AChBP complex [Ac-AChBP and the tested ligand (α-Bungarotoxin)] painted condition.

*Note: Additional ligands, including choline, nicotine, and amyloid β-Protein (1-42), can also be tested.*

2. Prepare protein complexes in solution at 1:10 molar ratio of Ac-AChBP:ligand. For a 65 μL total solution, for example, prepare:

2.94 μL of Ac-AChBP

1.60 μL of α-Bungarotoxin

61.26 μL of 1× PBS

For control samples that do not contain a ligand, bring the final volume up to 65 μL with 1× PBS.

3. Place the protein paint complex on a rotator and incubate at room temperature for 1 h with gentle mixing at 20 rpm.

*Note: Ensure that the solution is mixed well during the incubation.*

4. Prepare the appropriate number of Sephadex columns as samples. Give each column a sharp downward shake to ensure that the resin moves to the bottom of the column. Remove the cap and place the column in a 2 mL Eppendorf collection tube.

5. Using a 20–200 μL pipette, remove any remaining Sephadex gel from inside of the cap and place it in the column.

6. Spin the Sephadex columns at 1,000× *g* for 1 min.

*Note: Make sure there are no air pockets within the column as this might interfere with dye filtration.*

7. Add 400 μL of MilliQ water and let sit at room temperature until the protein paint complex incubation step (step B1.3) is complete.

**B2. Protein paint experiment**

1. Add 5 μL of diazo dye (5 mg/mL, 1 mg in 200 μL of PBS, prepared fresh) to each painted protein sample. Add 5 μL of 1× PBS to each sample that will be unpainted.

2. Incubate samples for 30 min at room temperature on a rotator.

3. Spin all columns at 1,000× *g* for 1 min. Add 5 μL of 5 mg/mL NHS ester dye to the painted protein samples. Add 5 μL of DMSO to the unpainted sample.

*Note: The volume must be consistent across the painted and unpainted groups during the entire experiment.*

4. Place all samples on a tube rotator and incubate for 30 min at room temperature.

5. During the incubation period, add 400 μL of MilliQ water to each Sephadex column and centrifuge at 1,000× *g* for 2 min.

6. Remove the Sephadex columns from the 2 mL tubes and place in new labeled Eppendorf Lo-Bind 1.5 mL tubes. Ensure there are no air pockets or cracks in the resin before adding samples. If these are present, repeat the previous step by adding MilliQ water and spinning the columns once again at the same settings.

7. When the incubation is complete, transfer the entire 75 μL of sample from each tube to the appropriate Sephadex column.

*Note: Carefully add the sample in the middle and avoid touching the resin with your pipette tip.*

8. Centrifuge all samples at 1,000× *g* for 1 min. Throw away columns and retain the flowthrough.

**B3. Denature proteins and add pre-digestion control**

1. Add 0.8 μL of 250 μg/mL lysozyme stock to each sample.

2. Add 25 μL of 8 M urea to each sample, for a final volume of 100 μL and a final urea concentration of 2M.

3. Add 1.1 μL of 1 M DTT to each sample, for a final volume of 101.1 μL and a final DTT concentration of 10mM.

4. Incubate samples at 37 °C for 30 min using a water bath.

5. Add 12 μL of iodoacetamide to each sample, for a final volume of 113.1 μL and a final iodoacetamide concentration of 50 mM.

6. Incubate samples at room temperature in a dark place for at least 15 min.

**B4. Trypsin digestion of protein complex**

1. Add the following reagents according to the recipe below:

86.32 μL of MilliQ H_2_O (ultrapure water)

22 μL of ammonium bicarbonate

0.58 μL of trypsin*

*If your calculations for a 1:10 trypsin ratio result in a trypsin volume less than 0.5 μL, just add 0.5 μL.

*This brings the final sample volume to 222 μL, final urea concentration to under 1M, ammonium bicarbonate to 50mM, and trypsin to 1:10 ratio.*

2. Allow samples to digest overnight in a hot water bath at 37 °C.

3. Halt digestion by adding 5 μL of acetic acid, ~2% acetic acid in the final sample (or you can skip straight to preparing the C-18 columns).

4. Store samples at -20 °C until they can be prepped for mass spectrometry.

**B5. Prepare C-18 columns**

1. Thaw samples and add 75 μL of sample buffer to each sample.

2. Centrifuge samples at 1,000× *g* for 1 min. For technical replicates, divide each sample into two 150 μL aliquots in protein Lo-Bind 1.5 mL Eppendorf tubes. Alternatively, pass all 150 μL of sample through the column at one time.

3. Follow the C-18 spin column manufacturer’s directions to remove salt from samples.

4. For the final spin column step, elute each sample into a clean 1.5 mL tube.

5. Dry samples under nitrogen at 40 °C for roughly 10 min. Monitor closely to ensure no sample is blown out of the tube. Place dried peptides at -20 °C until mass spectrometry analysis can be performed.

## Data analysis

1. LC/ESI MS: MS analysis was conducted in data-dependent mode with one full MS scan (60,000 resolving power) followed by MS/MS scans that target the top 20 abundant molecular ions fragmented by higher collisional dissociation (HCD). Tandem mass spectra were searched using Proteome Discover version 2.3 with SEQUEST against the NCBI *E. coli* databases and custom databases containing the recombinant protein sequence. Use a false discovery rate of 1% as the cutoff value for peptide spectrum matches (PSMs). In addition, label-free quantification via the use of the Minora algorithm within Proteome Discoverer can be applied to calculate protein abundances as described [10]. Peptides with less than two PSMs should be excluded from data analysis.

*Note: Additional mass spectrometry parameters (e.g., methionine oxidation detection) are provided in Graur et al. [6].*

2. Painted-to-unpainted abundance ratio measures and binding site validation:

Once data from at least three biological replicates is obtained, calculate separate abundance ratios for each identified peptide in the assay: the control ratio and the experimental ratio. For the control ratio, divide the peptide fragment's abundance reading in the painted Ac-AChBP sample by its abundance in the unpainted Ac-AChBP sample. For the experimental ratio, divide the peptide fragment's abundance reading in the painted Ac-AChBP + ligand sample by its abundance in the unpainted Ac-AChBP sample. If a ratio is less than 0.01, assign it a value of 0.01. Exclude peptide fragments with a control ratio greater than 0.25, which is likely due to dye inaccessibility. Perform a student’s t-test on the control and experimental ratios from all biological replicates to identify statistically significant peptide hits (p < 0.05).

*Note: Peptide cleavage products of trypsin and chymotrypsin digestion can yield some peptide regions overlap. This is advantageous in mapping out various segments of the AChBP that may participate in ligand binding.*

**Figure 3.**
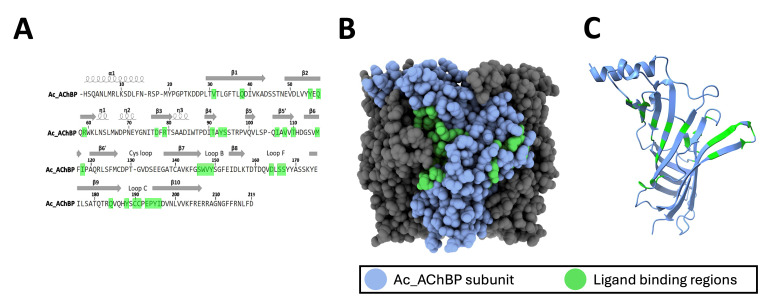
Analysis of protein interaction sites within Ac-AChBP. A. The sequence of the Ac-AChBP (UniProt; Q8WSF8; 1–219) showing the location of structural residues (α helix, β sheet) as well as ligand binding sites (green). B–C. ChimeraX rendering of the Ac-AChBP pentamer (B) showing the location of ligand binding sites (green) within an individual subunit (blue) (C).

3. Visualization of the identified protein interaction sites:

According to the Protein Data Bank (PDB), the Ac-AChBP structure can be found in several ligand-bound states including the apo state (PDB ID:2BYN), in complex with epibatidine (PDB ID:2BYQ), or in complex α-bungarotoxin (PDB ID:7KOO). Protein structures can be visualized with software packages such as ChimeraX [11], and putative regions of ligand binding within the protein sequence can be identified from peptides obtained using the protein paint experiment. ChimeraX and other protein structural modeling tools are useful for visualizing regions involved in protein binding within individual subunits as well as the pentameric structure of the Ac-AChBP (Figure 3). This information can be used to determine the proximity of residues involved in ligand binding as well as the overall topology of the protein binding region identified in the protein paint MS assay.

For a more detailed analysis, see Graur et al. [6].

## Validation of protocol

This protocol has been used and validated across various experiments in the following research articles:

- Graur et al. [6]. Protein Painting Mass Spectrometry in the Discovery of Interaction Sites within the Acetylcholine Binding Protein. *ACS Chemical Neuroscience*.
- Haymond et al. [12]. Protein painting, an optimized MS-based technique, reveals functionally relevant interfaces of the PD-1/PD-L1 complex and the YAP2/ZO-1 complex. *The Journal of Biological Chemistry*.
- Luchini et al. [8]. Protein painting reveals solvent-excluded drug targets hidden within native protein-protein interfaces. *Nature Communications*.

## General notes and troubleshooting

**Troubleshooting**

**Problem 1:** Ac-AChBP protein production yield is too low for the protein paint experiment.

Possible cause: Low protein synthesis within BL21 cells.

Solution: Repeat the protein induction protocol and pool two (or more) post-induction cell lysate solutions prior to the affinity purification step. This should increase the total amount of Ac-AChBP available for the study.

**Problem 2:** Peptide ratio for painted-to-unpainted peptides is not significant.

Possible cause: Ligand concentration may not be optimal.

Solution: Calculate the appropriate ligand concentration that would saturate the binding site.

**Problem 3:** Peptide fragments identified by MS are too long or too short.

Possible cause: Digestion with enzymes such as trypsin may create tryptic peptide fragments of various lengths depending on the amino acid composition of the full-length protein. In some cases, peptide fragments may therefore be too long or too short for meaningful analysis within the protein paint study.

Solution: Prior to the protein paint experiment, in silico tools such as Peptide Cutter can be used to predict the size and location of enzymatic digestion and peptide fragments generated by peptidase cleavage with certain enzymes. In addition, in some experiments, it may be best to use more than one enzyme for protein digestion.
